# Bis(4-amino­benzoato)-κ^2^ *O*,*O*′;κ*O*-(2,2′-bipyridine-κ^2^ *N*,*N*′)zinc

**DOI:** 10.1107/S1600536811039389

**Published:** 2011-09-30

**Authors:** Miao-Ling Huang, Ling-Ling Wang

**Affiliations:** a Department of Chemistry and Science of Life, Quanzhou Normal University, Fujian 362000, People’s Republic of China

## Abstract

In the title complex, [Zn(C_7_H_6_NO_2_)_2_(C_10_H_8_N_2_)], the Zn^II^ cation is coordinated by two amino­benzoate anions and one 2,2′-bipyridine ligand in a distorted trigonal–bipyramidal geometry. The carboxyl­ate group of one aminobenzoate anion coordinates to the Zn^II^ cation in a monodentate manner, whereas the carboxyl­ate group of the other amino­benzoate anion chelates the Zn cation with different Zn—O bond lengths. Inter­molecular N—H⋯N and N—H⋯O hydrogen bonding is present in the crystal structure.

## Related literature

For applications of Zn complexes, see: Chohan & Naseer (2007 ▶); Huang *et al.* (2006 ▶); Ispir *et al.* (2006 ▶); Lo *et al.* (2007 ▶); Maria *et al.* (1996 ▶). For a related structure, see: Wang *et al.* (2005 ▶).
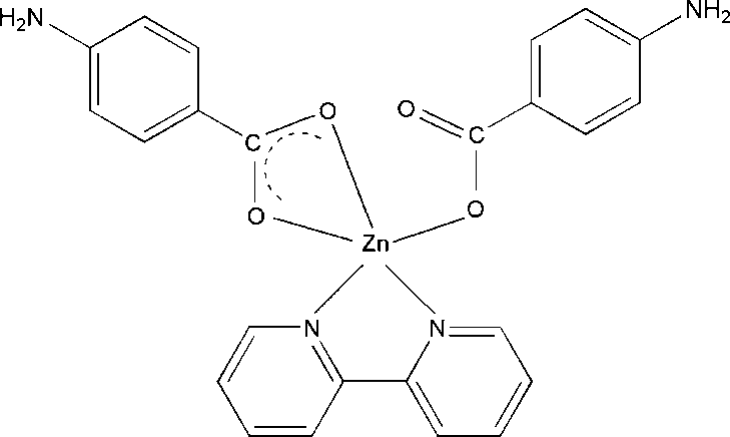


## Experimental

### 

#### Crystal data

[Zn(C_7_H_6_NO_2_)_2_(C_10_H_8_N_2_)]
*M* *_r_* = 493.81
Triclinic, 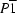

*a* = 7.9499 (14) Å
*b* = 10.7281 (19) Å
*c* = 13.905 (2) Å
α = 80.499 (2)°
β = 80.921 (2)°
γ = 70.538 (2)°
*V* = 1096.1 (3) Å^3^
*Z* = 2
Mo *K*α radiation
μ = 1.16 mm^−1^
*T* = 295 K
0.42 × 0.23 × 0.08 mm

#### Data collection

Bruker SMART 1000 CCD area-detector diffractometer
Absorption correction: multi-scan (*SADABS*; Bruker, 2001 ▶) *T*_min_ = 0.643, *T*_max_ = 0.914
8223 measured reflections
4058 independent reflections
3419 reflections with *I* > 2σ(*I*)
*R*_int_ = 0.024

#### Refinement

*R*[*F* ^2^ > 2σ(*F* ^2^)] = 0.035
*wR*(*F* ^2^) = 0.090
*S* = 1.07
4058 reflections
298 parameters
H-atom parameters constrained
Δρ_max_ = 0.31 e Å^−3^
Δρ_min_ = −0.31 e Å^−3^

### 

Data collection: *SMART* (Bruker, 2007 ▶); cell refinement: *SAINT* (Bruker, 2007 ▶); data reduction: *SAINT*; program(s) used to solve structure: *SHELXTL* (Sheldrick, 2008 ▶); program(s) used to refine structure: *SHELXTL*; molecular graphics: *SHELXTL* (Sheldrick, 2008 ▶); software used to prepare material for publication: *SHELXTL*.

## Supplementary Material

Crystal structure: contains datablock(s) global, I. DOI: 10.1107/S1600536811039389/xu5334sup1.cif

Structure factors: contains datablock(s) I. DOI: 10.1107/S1600536811039389/xu5334Isup2.hkl

Additional supplementary materials:  crystallographic information; 3D view; checkCIF report

## Figures and Tables

**Table 1:** Selected bond lengths (Å)

Zn1—O1	1.9269 (18)
Zn1—O3	1.9704 (18)
Zn1—O4	2.395 (2)
Zn1—N3	2.124 (2)
Zn1—N4	2.088 (2)

**Table 2:** Hydrogen-bond geometry (Å, °)

*D*—H⋯*A*	*D*—H	H⋯*A*	*D*⋯*A*	*D*—H⋯*A*
N1—H1*D*⋯N2^i^	0.88	2.33	3.202 (4)	169
N1—H1*E*⋯O2^ii^	0.88	2.36	3.181 (4)	155
N2—H2*E*⋯O2^iii^	0.88	2.28	3.116 (4)	159

Symmetry codes: (i) 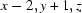; (ii) 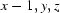; (iii) 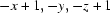.

**Fig. 1.**
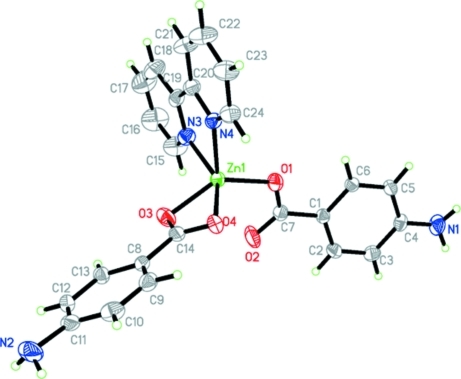
The ORTEP drawing of the title compound (I). Displacement ellipsoids are drawn at 30% probability level.

**Fig. 2.**
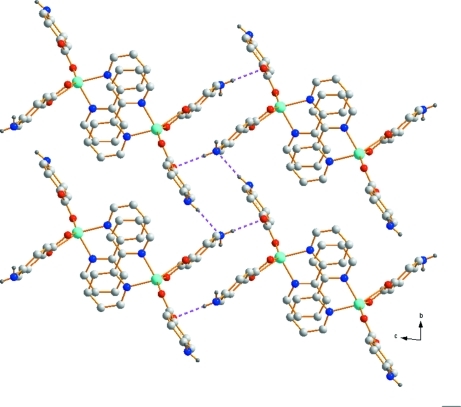
Projection showing the two-dimensional structure formed by H-bonding interaction of the compound (I).

## supplementary crystallographic information

### Comment

In recent decades, zinc complexes have received much attention because of their interesting with biological ligands to generate stable mixed coordinated complexes, which play a key role in life process such as anti-cancer, antiseptic and anti-inflammatory (Ispir *et al.*, 2006; Chohan & Naseer, 2007; Huang *et al.*, 2006; Lo *et al.*, 2007). 4-Aminobenzoic acid as an important part in the folic acid, which is a constituent of the vitamin B complex and is found in animal and plant tissues, has been shown to be a growth factor in certain microorganisms (Maria *et al.*, 1996). In order to extend further the study of 4-aminobenzoic acid ligand coordinate to zinc ion, we synthesized the title complex and determined the crystal structure.

The asymmetric unit of 1 contains one zinc cation, two 4-aminobenzoic ion, and one 2,2'-bipyridine molecule. The Zn(II) atom is five-coordinated, forming a distorted trigonal-bipyramidal (Table 1). In the coordination polyhedron, the equatorial plane is occupied by two O(O1,O3) atoms from different 4-aba and one N(N4) atom from 2,2'-bipyridine, at the apex is situated one O(O4) atom from 4-aba and one N(N3) atom from 2,2'-bipyridine (Fig 1). The Zn(II) center is coordinated by two types of 4-aba: one behaves in an unsymmetrical chelating mode [Zn—O(3) 1.9704 (18) and Zn—O(4) 2.395 (2) Å]; the other acts as a monodentate ligand through one carboxylate oxygen atom [Zn—O(1) 1.9269 (18) and Zn—O(2) 2.841 Å], which is similar to previously reported complex {[Zn_2_(4,4'-bipy)_2_(4-aba)_4_](H_2_O)_5_} (Wang *et al.*, 2005). The dihedral angle between phenyl rings of the two 4-aba is 81.83 (7)°.

Hydrogen bonds are observed between the molecules in the crystal structure (Table 2).

### Experimental

An aqueous solution (5 ml) of ZnC_4_H_6_O_4_.2H_2_O (1 mmol) was added slowly to a mixed solution of 4-aminobenzoic acid (1.5 mmol) in H_2_O (5 ml) and 2,2'-bipyridine (1 mmol) in ethanol (95%, 5 ml). After refluxing for 3 h, the mixture was filtered off while hot. The colourless single crystals suitable for X-ray analysis were obtained by slow evaporation of the above filtrate at room temperature after a month.

### Refinement

H atoms were placed geometrically and treated as riding, C—H = 0.93 and N—H = 0.88 Å, U_iso_(H) = 1.2U_eq_(C) and 1.5U_eq_(N).

### Crystal data

**Table:**

[Zn(C_7_H_6_NO_2_)_2_(C_10_H_8_N_2_)]	*Z* = 2
*M**_r_* = 493.81	*F*(000) = 508
Triclinic, *P*1	*D*_x_ = 1.496 Mg m^−^^3^
Hall symbol: -P 1	Mo *K*α radiation, λ = 0.71073 Å
*a* = 7.9499 (14) Å	Cell parameters from 2868 reflections
*b* = 10.7281 (19) Å	θ = 2.4–24.9°
*c* = 13.905 (2) Å	µ = 1.16 mm^−^^1^
α = 80.499 (2)°	*T* = 295 K
β = 80.921 (2)°	Block, colourless
γ = 70.538 (2)°	0.42 × 0.23 × 0.08 mm
*V* = 1096.1 (3) Å^3^	

### Data collection

**Table:**

Bruker SMART 1000 CCD area-detector diffractometer	4058 independent reflections
Radiation source: fine-focus sealed tube	3419 reflections with *I* > 2σ(*I*)
graphite	*R*_int_ = 0.024
φ and ω scans	θ_max_ = 25.5°, θ_min_ = 2.4°
Absorption correction: multi-scan (*SADABS*; Bruker, 2001)	*h* = −9→9
*T*_min_ = 0.643, *T*_max_ = 0.914	*k* = −12→12
8223 measured reflections	*l* = −16→16

### Refinement

**Table:**

Refinement on *F*^2^	Primary atom site location: structure-invariant direct methods
Least-squares matrix: full	Secondary atom site location: difference Fourier map
*R*[*F*^2^ > 2σ(*F*^2^)] = 0.035	Hydrogen site location: inferred from neighbouring sites
*wR*(*F*^2^) = 0.090	H-atom parameters constrained
*S* = 1.07	*w* = 1/[σ^2^(*F*_o_^2^) + (0.0411*P*)^2^ + 0.2825*P*] where *P* = (*F*_o_^2^ + 2*F*_c_^2^)/3
4058 reflections	(Δ/σ)_max_ < 0.001
298 parameters	Δρ_max_ = 0.31 e Å^−^^3^
0 restraints	Δρ_min_ = −0.31 e Å^−^^3^

### Special details

**Table:**

Geometry. All e.s.d.'s (except the e.s.d. in the dihedral angle between two l.s. planes) are estimated using the full covariance matrix. The cell e.s.d.'s are taken into account individually in the estimation of e.s.d.'s in distances, angles and torsion angles; correlations between e.s.d.'s in cell parameters are only used when they are defined by crystal symmetry. An approximate (isotropic) treatment of cell e.s.d.'s is used for estimating e.s.d.'s involving l.s. planes.
Refinement. Refinement of *F*^2^ against ALL reflections. The weighted *R*-factor *wR* and goodness of fit *S* are based on *F*^2^, conventional *R*-factors *R* are based on *F*, with *F* set to zero for negative *F*^2^. The threshold expression of *F*^2^ > σ(*F*^2^) is used only for calculating *R*-factors(gt) *etc*. and is not relevant to the choice of reflections for refinement. *R*-factors based on *F*^2^ are statistically about twice as large as those based on *F*, and *R*- factors based on ALL data will be even larger.

### Fractional atomic coordinates and isotropic or equivalent isotropic displacement parameters (Å^2^)

**Table:**

	*x*	*y*	*z*	*U*_iso_*/*U*_eq_	
Zn1	0.14918 (4)	0.12380 (3)	0.19516 (2)	0.04021 (12)	
O1	−0.1044 (2)	0.2151 (2)	0.21855 (14)	0.0528 (5)	
O2	−0.0311 (3)	0.3661 (2)	0.27669 (18)	0.0732 (7)	
O3	0.3785 (3)	0.10054 (19)	0.24420 (15)	0.0558 (5)	
O4	0.2405 (3)	−0.0296 (2)	0.33755 (15)	0.0663 (6)	
N1	−0.8907 (3)	0.5767 (3)	0.3483 (2)	0.0678 (7)	
H1D	−0.9291	0.6259	0.3971	0.102*	
H1E	−0.9611	0.5323	0.3389	0.102*	
N2	1.0034 (4)	−0.2214 (3)	0.5101 (2)	0.0788 (9)	
H2D	1.0548	−0.1604	0.5101	0.118*	
H2E	0.9810	−0.2610	0.5693	0.118*	
N3	0.2165 (3)	0.1976 (2)	0.04781 (16)	0.0502 (6)	
N4	0.1805 (3)	−0.0382 (2)	0.12124 (15)	0.0419 (5)	
C1	−0.3406 (3)	0.3914 (2)	0.28122 (17)	0.0362 (5)	
C2	−0.4024 (4)	0.4919 (3)	0.3417 (2)	0.0488 (7)	
H2	−0.3201	0.5185	0.3670	0.059*	
C3	−0.5831 (4)	0.5529 (3)	0.3648 (2)	0.0537 (7)	
H3	−0.6211	0.6196	0.4057	0.064*	
C4	−0.7100 (3)	0.5157 (3)	0.3275 (2)	0.0444 (6)	
C5	−0.6484 (3)	0.4166 (2)	0.26597 (19)	0.0417 (6)	
H5	−0.7303	0.3914	0.2391	0.050*	
C6	−0.4677 (3)	0.3554 (2)	0.24420 (18)	0.0375 (5)	
H6	−0.4297	0.2882	0.2036	0.045*	
C7	−0.1451 (3)	0.3227 (3)	0.25832 (18)	0.0423 (6)	
C8	0.5359 (3)	−0.0506 (2)	0.36920 (17)	0.0368 (5)	
C9	0.5508 (4)	−0.1572 (3)	0.44177 (19)	0.0479 (7)	
H9	0.4560	−0.1919	0.4589	0.058*	
C10	0.7028 (4)	−0.2133 (3)	0.4894 (2)	0.0560 (8)	
H10	0.7105	−0.2861	0.5372	0.067*	
C11	0.8444 (4)	−0.1614 (3)	0.4663 (2)	0.0495 (7)	
C12	0.8290 (3)	−0.0533 (3)	0.3952 (2)	0.0474 (6)	
H12	0.9222	−0.0167	0.3796	0.057*	
C13	0.6791 (3)	0.0007 (3)	0.34731 (18)	0.0416 (6)	
H13	0.6723	0.0730	0.2992	0.050*	
C14	0.3746 (3)	0.0096 (3)	0.3162 (2)	0.0456 (6)	
C15	0.2298 (5)	0.3190 (3)	0.0162 (3)	0.0771 (10)	
H15	0.2011	0.3809	0.0607	0.093*	
C16	0.2848 (7)	0.3549 (4)	−0.0801 (3)	0.1001 (14)	
H16	0.2920	0.4402	−0.1006	0.120*	
C17	0.3286 (6)	0.2642 (5)	−0.1448 (3)	0.0988 (14)	
H17	0.3678	0.2865	−0.2100	0.119*	
C18	0.3146 (5)	0.1395 (4)	−0.1135 (2)	0.0714 (9)	
H18	0.3437	0.0765	−0.1571	0.086*	
C19	0.2563 (3)	0.1089 (3)	−0.01574 (19)	0.0479 (7)	
C20	0.2330 (3)	−0.0219 (3)	0.02410 (19)	0.0461 (6)	
C21	0.2580 (5)	−0.1217 (3)	−0.0330 (2)	0.0702 (9)	
H21	0.2929	−0.1097	−0.1000	0.084*	
C22	0.2307 (6)	−0.2389 (4)	0.0103 (3)	0.0783 (10)	
H22	0.2470	−0.3069	−0.0274	0.094*	
C23	0.1794 (5)	−0.2557 (3)	0.1088 (2)	0.0673 (9)	
H23	0.1607	−0.3348	0.1390	0.081*	
C24	0.1561 (4)	−0.1535 (3)	0.1622 (2)	0.0534 (7)	
H24	0.1221	−0.1648	0.2293	0.064*	

### Atomic displacement parameters (Å^2^)

**Table:**

	*U*^11^	*U*^22^	*U*^33^	*U*^12^	*U*^13^	*U*^23^
Zn1	0.03267 (17)	0.04633 (19)	0.03920 (18)	−0.00652 (13)	−0.00538 (12)	−0.00961 (13)
O1	0.0335 (9)	0.0589 (12)	0.0619 (12)	−0.0044 (9)	−0.0064 (8)	−0.0168 (10)
O2	0.0385 (11)	0.0841 (16)	0.1075 (19)	−0.0255 (11)	−0.0096 (11)	−0.0263 (14)
O3	0.0480 (11)	0.0515 (12)	0.0688 (13)	−0.0078 (9)	−0.0261 (10)	−0.0074 (10)
O4	0.0385 (11)	0.1067 (18)	0.0642 (13)	−0.0291 (11)	−0.0019 (9)	−0.0299 (12)
N1	0.0407 (13)	0.0650 (17)	0.092 (2)	−0.0066 (12)	0.0100 (13)	−0.0317 (15)
N2	0.0660 (18)	0.0786 (19)	0.0765 (19)	0.0202 (15)	−0.0384 (15)	−0.0286 (15)
N3	0.0525 (14)	0.0509 (14)	0.0436 (13)	−0.0125 (11)	−0.0062 (11)	−0.0024 (11)
N4	0.0386 (11)	0.0477 (13)	0.0367 (12)	−0.0080 (10)	−0.0058 (9)	−0.0080 (10)
C1	0.0333 (12)	0.0379 (13)	0.0360 (13)	−0.0109 (10)	−0.0050 (10)	−0.0006 (11)
C2	0.0466 (16)	0.0530 (17)	0.0544 (17)	−0.0212 (13)	−0.0054 (13)	−0.0165 (13)
C3	0.0538 (17)	0.0496 (17)	0.0607 (18)	−0.0152 (14)	0.0040 (14)	−0.0272 (14)
C4	0.0370 (13)	0.0389 (14)	0.0521 (16)	−0.0087 (11)	0.0046 (12)	−0.0069 (12)
C5	0.0346 (13)	0.0412 (14)	0.0519 (15)	−0.0135 (11)	−0.0053 (11)	−0.0092 (12)
C6	0.0361 (13)	0.0355 (13)	0.0392 (13)	−0.0078 (10)	−0.0030 (10)	−0.0089 (11)
C7	0.0371 (14)	0.0484 (16)	0.0369 (14)	−0.0096 (12)	−0.0068 (11)	0.0012 (12)
C8	0.0330 (12)	0.0403 (14)	0.0373 (13)	−0.0090 (10)	−0.0007 (10)	−0.0133 (11)
C9	0.0513 (16)	0.0508 (16)	0.0463 (15)	−0.0246 (13)	0.0056 (13)	−0.0115 (13)
C10	0.074 (2)	0.0433 (16)	0.0413 (15)	−0.0070 (15)	−0.0095 (14)	0.0011 (12)
C11	0.0436 (15)	0.0522 (16)	0.0433 (15)	0.0072 (13)	−0.0117 (12)	−0.0200 (13)
C12	0.0339 (13)	0.0607 (18)	0.0493 (16)	−0.0137 (13)	−0.0017 (12)	−0.0166 (14)
C13	0.0380 (14)	0.0432 (14)	0.0421 (14)	−0.0116 (11)	−0.0056 (11)	−0.0025 (11)
C14	0.0349 (14)	0.0535 (17)	0.0504 (16)	−0.0071 (12)	−0.0044 (12)	−0.0270 (14)
C15	0.111 (3)	0.059 (2)	0.064 (2)	−0.034 (2)	−0.013 (2)	0.0027 (17)
C16	0.150 (4)	0.081 (3)	0.072 (3)	−0.056 (3)	−0.009 (3)	0.021 (2)
C17	0.128 (4)	0.107 (3)	0.055 (2)	−0.048 (3)	0.008 (2)	0.013 (2)
C18	0.083 (2)	0.077 (2)	0.0436 (17)	−0.0196 (19)	0.0055 (16)	−0.0039 (16)
C19	0.0380 (14)	0.0542 (17)	0.0414 (15)	−0.0035 (12)	−0.0025 (11)	−0.0029 (13)
C20	0.0405 (14)	0.0507 (16)	0.0413 (15)	−0.0034 (12)	−0.0083 (11)	−0.0093 (12)
C21	0.095 (3)	0.065 (2)	0.0431 (17)	−0.0107 (19)	−0.0063 (17)	−0.0166 (15)
C22	0.114 (3)	0.058 (2)	0.063 (2)	−0.017 (2)	−0.016 (2)	−0.0232 (17)
C23	0.090 (2)	0.0486 (18)	0.066 (2)	−0.0206 (17)	−0.0171 (18)	−0.0092 (16)
C24	0.0610 (18)	0.0525 (17)	0.0464 (16)	−0.0170 (14)	−0.0079 (13)	−0.0054 (13)

### Geometric parameters (Å, °)

**Table:**

Zn1—O1	1.9269 (18)	C6—H6	0.9300
Zn1—O3	1.9704 (18)	C8—C9	1.381 (4)
Zn1—O4	2.395 (2)	C8—C13	1.394 (3)
Zn1—N3	2.124 (2)	C8—C14	1.481 (4)
Zn1—N4	2.088 (2)	C9—C10	1.377 (4)
Zn1—C14	2.521 (3)	C9—H9	0.9300
O1—C7	1.284 (3)	C10—C11	1.387 (4)
O2—C7	1.223 (3)	C10—H10	0.9300
O3—C14	1.282 (3)	C11—C12	1.380 (4)
O4—C14	1.249 (3)	C12—C13	1.364 (3)
N1—C4	1.372 (3)	C12—H12	0.9300
N1—H1D	0.8818	C13—H13	0.9300
N1—H1E	0.8820	C15—C16	1.375 (5)
N2—C11	1.397 (3)	C15—H15	0.9300
N2—H2D	0.8798	C16—C17	1.358 (6)
N2—H2E	0.8825	C16—H16	0.9300
N3—C19	1.332 (4)	C17—C18	1.372 (5)
N3—C15	1.338 (4)	C17—H17	0.9300
N4—C24	1.335 (3)	C18—C19	1.388 (4)
N4—C20	1.350 (3)	C18—H18	0.9300
C1—C2	1.388 (4)	C19—C20	1.481 (4)
C1—C6	1.388 (3)	C20—C21	1.381 (4)
C1—C7	1.490 (3)	C21—C22	1.371 (5)
C2—C3	1.375 (4)	C21—H21	0.9300
C2—H2	0.9300	C22—C23	1.367 (5)
C3—C4	1.396 (4)	C22—H22	0.9300
C3—H3	0.9300	C23—C24	1.371 (4)
C4—C5	1.386 (4)	C23—H23	0.9300
C5—C6	1.373 (3)	C24—H24	0.9300
C5—H5	0.9300		
			
O1—Zn1—O3	141.27 (9)	C13—C8—C14	120.3 (2)
O1—Zn1—N4	107.51 (8)	C10—C9—C8	121.5 (3)
O3—Zn1—N4	108.58 (8)	C10—C9—H9	119.2
O1—Zn1—N3	103.23 (9)	C8—C9—H9	119.2
O3—Zn1—N3	97.43 (9)	C9—C10—C11	120.2 (3)
N4—Zn1—N3	78.22 (9)	C9—C10—H10	119.9
O1—Zn1—O4	108.31 (8)	C11—C10—H10	119.9
O3—Zn1—O4	59.37 (8)	C12—C11—C10	118.6 (2)
N4—Zn1—O4	88.61 (8)	C12—C11—N2	120.4 (3)
N3—Zn1—O4	148.23 (8)	C10—C11—N2	120.9 (3)
O1—Zn1—C14	129.59 (8)	C13—C12—C11	121.0 (3)
O3—Zn1—C14	30.11 (8)	C13—C12—H12	119.5
N4—Zn1—C14	98.66 (8)	C11—C12—H12	119.5
N3—Zn1—C14	124.19 (9)	C12—C13—C8	121.2 (2)
O4—Zn1—C14	29.29 (8)	C12—C13—H13	119.4
C7—O1—Zn1	114.85 (16)	C8—C13—H13	119.4
C14—O3—Zn1	99.43 (16)	O4—C14—O3	120.2 (3)
C14—O4—Zn1	80.92 (18)	O4—C14—C8	121.8 (3)
C4—N1—H1D	119.3	O3—C14—C8	118.0 (2)
C4—N1—H1E	115.8	O4—C14—Zn1	69.79 (16)
H1D—N1—H1E	115.8	O3—C14—Zn1	50.46 (12)
C11—N2—H2D	108.4	C8—C14—Zn1	167.2 (2)
C11—N2—H2E	110.2	N3—C15—C16	121.9 (4)
H2D—N2—H2E	113.1	N3—C15—H15	119.1
C19—N3—C15	119.3 (3)	C16—C15—H15	119.1
C19—N3—Zn1	114.43 (18)	C17—C16—C15	119.1 (4)
C15—N3—Zn1	126.2 (2)	C17—C16—H16	120.5
C24—N4—C20	119.2 (2)	C15—C16—H16	120.5
C24—N4—Zn1	125.67 (18)	C16—C17—C18	119.6 (3)
C20—N4—Zn1	115.14 (18)	C16—C17—H17	120.2
C2—C1—C6	117.6 (2)	C18—C17—H17	120.2
C2—C1—C7	121.3 (2)	C17—C18—C19	118.9 (3)
C6—C1—C7	121.1 (2)	C17—C18—H18	120.5
C3—C2—C1	121.3 (2)	C19—C18—H18	120.5
C3—C2—H2	119.4	N3—C19—C18	121.2 (3)
C1—C2—H2	119.4	N3—C19—C20	116.1 (2)
C2—C3—C4	120.8 (2)	C18—C19—C20	122.7 (3)
C2—C3—H3	119.6	N4—C20—C21	120.7 (3)
C4—C3—H3	119.6	N4—C20—C19	115.9 (2)
N1—C4—C5	120.3 (3)	C21—C20—C19	123.4 (3)
N1—C4—C3	121.7 (3)	C22—C21—C20	119.3 (3)
C5—C4—C3	118.0 (2)	C22—C21—H21	120.4
C6—C5—C4	120.8 (2)	C20—C21—H21	120.4
C6—C5—H5	119.6	C23—C22—C21	119.9 (3)
C4—C5—H5	119.6	C23—C22—H22	120.0
C5—C6—C1	121.6 (2)	C21—C22—H22	120.0
C5—C6—H6	119.2	C22—C23—C24	118.5 (3)
C1—C6—H6	119.2	C22—C23—H23	120.7
O2—C7—O1	122.4 (2)	C24—C23—H23	120.7
O2—C7—C1	122.0 (3)	N4—C24—C23	122.4 (3)
O1—C7—C1	115.6 (2)	N4—C24—H24	118.8
C9—C8—C13	117.5 (2)	C23—C24—H24	118.8
C9—C8—C14	122.2 (2)		
			
O3—Zn1—O1—C7	26.2 (3)	C10—C11—C12—C13	−0.9 (4)
N4—Zn1—O1—C7	−175.82 (17)	N2—C11—C12—C13	175.6 (2)
N3—Zn1—O1—C7	−94.20 (19)	C11—C12—C13—C8	0.6 (4)
O4—Zn1—O1—C7	89.73 (19)	C9—C8—C13—C12	0.6 (4)
C14—Zn1—O1—C7	66.4 (2)	C14—C8—C13—C12	−179.9 (2)
O1—Zn1—O3—C14	83.1 (2)	Zn1—O4—C14—O3	3.2 (2)
N4—Zn1—O3—C14	−74.76 (17)	Zn1—O4—C14—C8	−173.9 (2)
N3—Zn1—O3—C14	−154.77 (16)	Zn1—O3—C14—O4	−3.9 (3)
O4—Zn1—O3—C14	2.02 (14)	Zn1—O3—C14—C8	173.36 (18)
O1—Zn1—O4—C14	−141.45 (15)	C9—C8—C14—O4	3.2 (4)
O3—Zn1—O4—C14	−2.07 (15)	C13—C8—C14—O4	−176.3 (2)
N4—Zn1—O4—C14	110.56 (16)	C9—C8—C14—O3	−174.0 (2)
N3—Zn1—O4—C14	45.8 (2)	C13—C8—C14—O3	6.5 (3)
O1—Zn1—N3—C19	−109.74 (19)	C9—C8—C14—Zn1	−150.2 (7)
O3—Zn1—N3—C19	103.24 (19)	C13—C8—C14—Zn1	30.3 (9)
N4—Zn1—N3—C19	−4.28 (19)	O1—Zn1—C14—O4	50.15 (19)
O4—Zn1—N3—C19	63.2 (3)	O3—Zn1—C14—O4	176.4 (3)
C14—Zn1—N3—C19	88.3 (2)	N4—Zn1—C14—O4	−71.23 (16)
O1—Zn1—N3—C15	73.6 (3)	N3—Zn1—C14—O4	−152.83 (15)
O3—Zn1—N3—C15	−73.4 (3)	O1—Zn1—C14—O3	−126.29 (17)
N4—Zn1—N3—C15	179.1 (3)	N4—Zn1—C14—O3	112.32 (16)
O4—Zn1—N3—C15	−113.5 (3)	N3—Zn1—C14—O3	30.7 (2)
C14—Zn1—N3—C15	−88.4 (3)	O4—Zn1—C14—O3	−176.4 (3)
O1—Zn1—N4—C24	−77.9 (2)	O1—Zn1—C14—C8	−153.8 (8)
O3—Zn1—N4—C24	87.8 (2)	O3—Zn1—C14—C8	−27.5 (8)
N3—Zn1—N4—C24	−178.2 (2)	N4—Zn1—C14—C8	84.8 (8)
O4—Zn1—N4—C24	30.9 (2)	N3—Zn1—C14—C8	3.2 (9)
C14—Zn1—N4—C24	58.5 (2)	O4—Zn1—C14—C8	156.1 (9)
O1—Zn1—N4—C20	103.27 (18)	C19—N3—C15—C16	−0.5 (5)
O3—Zn1—N4—C20	−91.02 (18)	Zn1—N3—C15—C16	176.0 (3)
N3—Zn1—N4—C20	2.95 (17)	N3—C15—C16—C17	−0.7 (7)
O4—Zn1—N4—C20	−147.95 (18)	C15—C16—C17—C18	1.0 (7)
C14—Zn1—N4—C20	−120.34 (18)	C16—C17—C18—C19	−0.2 (6)
C6—C1—C2—C3	0.6 (4)	C15—N3—C19—C18	1.3 (4)
C7—C1—C2—C3	−178.3 (2)	Zn1—N3—C19—C18	−175.6 (2)
C1—C2—C3—C4	−0.3 (4)	C15—N3—C19—C20	−178.2 (3)
C2—C3—C4—N1	−178.9 (3)	Zn1—N3—C19—C20	4.9 (3)
C2—C3—C4—C5	−0.6 (4)	C17—C18—C19—N3	−0.9 (5)
N1—C4—C5—C6	179.6 (2)	C17—C18—C19—C20	178.5 (3)
C3—C4—C5—C6	1.3 (4)	C24—N4—C20—C21	1.3 (4)
C4—C5—C6—C1	−1.1 (4)	Zn1—N4—C20—C21	−179.8 (2)
C2—C1—C6—C5	0.1 (4)	C24—N4—C20—C19	179.7 (2)
C7—C1—C6—C5	179.0 (2)	Zn1—N4—C20—C19	−1.4 (3)
Zn1—O1—C7—O2	2.4 (3)	N3—C19—C20—N4	−2.4 (3)
Zn1—O1—C7—C1	−177.28 (15)	C18—C19—C20—N4	178.1 (3)
C2—C1—C7—O2	−13.1 (4)	N3—C19—C20—C21	176.0 (3)
C6—C1—C7—O2	168.1 (3)	C18—C19—C20—C21	−3.5 (4)
C2—C1—C7—O1	166.6 (2)	N4—C20—C21—C22	−0.7 (5)
C6—C1—C7—O1	−12.2 (3)	C19—C20—C21—C22	−179.0 (3)
C13—C8—C9—C10	−1.5 (4)	C20—C21—C22—C23	0.0 (6)
C14—C8—C9—C10	179.0 (2)	C21—C22—C23—C24	0.1 (6)
C8—C9—C10—C11	1.2 (4)	C20—N4—C24—C23	−1.2 (4)
C9—C10—C11—C12	0.0 (4)	Zn1—N4—C24—C23	−180.0 (2)
C9—C10—C11—N2	−176.5 (2)	C22—C23—C24—N4	0.5 (5)

### Hydrogen-bond geometry (Å, °)

**Table:**

*D*—H···*A*	*D*—H	H···*A*	*D*···*A*	*D*—H···*A*
N1—H1D···N2^i^	0.88	2.33	3.202 (4)	169.
N1—H1E···O2^ii^	0.88	2.36	3.181 (4)	155.
N2—H2E···O2^iii^	0.88	2.28	3.116 (4)	159.

Symmetry codes: (i) *x*−2, *y*+1, *z*; (ii) *x*−1, *y*, *z*; (iii) −*x*+1, −*y*, −*z*+1.
